# Anterior Cervical Discectomy and Fusion Performed Using a CaO-SiO_2_-P_2_O_5_-B_2_O_3_ Bioactive Glass Ceramic or Polyetheretherketone Cage Filled with Hydroxyapatite/β-Tricalcium Phosphate: A Prospective Randomized Controlled Trial

**DOI:** 10.3390/jcm12124069

**Published:** 2023-06-15

**Authors:** Jiwon Park, Sang-Min Park, Dae-Woong Ham, Jae-Young Hong, Ho-Joong Kim, Jin S. Yeom

**Affiliations:** 1 Department of Orthopedic Surgery, Korea University Ansan Hospital, Ansan-si 15355, Republic of Korea; jwpark506@gmail.com (J.P.); osspine@korea.ac.kr (J.-Y.H.); 2 Spine Center and Department of Orthopedic Surgery, Seoul National University College of Medicine and Seoul National University Bundang Hospital, Seongnam 13620, Republic of Korea; grotyx@gmail.com (S.-M.P.); oshjkim@gmail.com (H.-J.K.); 3 Department of Orthopedic Surgery, Chung-Ang University Hospital, College of Medicine, Chung-Ang University, Seoul 06974, Republic of Korea; hamdgogo@gmail.com

**Keywords:** ACDF, cervical, fusion rate, glass ceramics, PEEK cage

## Abstract

A CaO-SiO_2_-P_2_O_5_-B_2_O_3_ bioactive glass-ceramic (BGS-7) spacer provides high mechanical stability, produces a chemical bond to the adjacent endplate, and facilitates fusion after spine surgery. This prospective, randomized, single-blind, non-inferiority trial aimed to evaluate the radiographic outcomes and clinical efficacy of anterior cervical discectomy and fusion (ACDF) using a BGS-7 spacer for treating cervical degenerative disorders. Thirty-six patients underwent ACDF using a BGS-7 spacer (Group N), and 40 patients underwent ACDF using polyetheretherketone (PEEK) cages filled with a mixture of hydroxyapatite (HA) and β-tricalcium phosphate (β-TCP) for the treatment of cervical degenerative disorders. The spinal fusion rate was assessed 12 months postoperatively using three-dimensional computed tomography (CT) and dynamic radiographs. Clinical outcomes included patient-reported outcome measures, visual analog scale scores for neck and arm pain, and scores from the neck disability index (NDI), European Quality of Life-5 Dimensions (EQ-5D), and 12-item Short Form Survey (SF-12v2). All participants were randomly assigned to undergo ACDF using either a BGS-7 spacer or PEEK cage filled with HA and β-TCP. The primary outcome was the fusion rate on CT scan image at 12 months after ACDF surgery based on a per-protocol strategy. Clinical outcomes and adverse events were also assessed. The 12-month fusion rates for the BGS-7 and PEEK groups based on CT scans were 81.8% and 74.4%, respectively, while those based on dynamic radiographs were 78.1% and 73.7%, respectively, with no significant difference between the groups. There were no significant differences in the clinical outcomes between the two groups. Neck pain, arm pain, NDI, EQ-5D, and SF-12v2 scores significantly improved postoperatively, with no significant differences between the groups. No adverse events were observed in either group. In ACDF surgery, the BGS-7 spacer showed similar fusion rates and clinical outcomes as PEEK cages filled with HA and β-TCP.

## 1. Introduction

Anterior cervical discectomy and fusion (ACDF) is a beneficial approach for surgically addressing cervical myelopathy or radiculopathy. An autogenous bone graft from the iliac crest is widely regarded as the optimal bone graft material for cervical interbody fusion. However, the application of autologous bone graft for ACDF is restricted due to the occurrence of donor site morbidity, which includes pain, infection, donor site fracture, hematoma, or meralgia paresthetica [1,2,3,4]. In this regard, the utilization of allografts has emerged as a viable substitute in this context. Nevertheless, complications associated with allografts continue to persist as issues, such as inadequate fusion rates and high graft subsidence or fracture [1,2,3,4]. To overcome these limitations, synthetic cage materials, namely titanium, carbon fibers, and polyetheretherketon (PEEK) have been implemented. Titanium and PEEK primarily offer structural support for interbody fusion. However, due to their inability to bind to the bone by itself, they necessitate the use of ‘filler’ bone substitutes to facilitate integration with the surrounding bone [4,5]. Various bone substitutes, such as demineralized bone matrix (DBM), beta-ticalcum phosphate (β-TCP), and hydroxyapatite (HA) have been utilized in order to replace the autogenous bone graft filler inside the cage [6,7]. 

Bioactive glass-ceramics, which have been introduced recently, are recognized for their ability to establish a chemical bond with bones and generate a carbohydroxy apatite layer [8,9]. The mechanical strength of bioactive glass-ceramics surpasses that of other bone substitutes, as evidenced by their high compressive and bending strength, according to previous studies [6,7,8,9]. In addition, it has been observed that CaO-SiO_2_-P_2_O_5_-B_2_O_3_ glass-ceramic (BGS-7), which is a form of bioactive glass-ceramic, exhibits the capability to stimulate the differentiation of human mesenchymal stem cells into osteoblasts. This property of BGS-7 has been found to enhance the process of osteointegration of bone-implants, thereby highlighting its potential in the field of spine surgery [9,10]. In a prior in vivo study, BGS-7 exhibited enhanced bone adhesion to neighboring bones in comparison to HA [10,11,12]. It has been suggested that BGS-7 is biocompatible, as evidenced by its lack of toxicity and adverse effects in a repeated intravenous toxicity study [13]. Clinically, the efficacy and safety of BGS-7 as an interbody spacer has been reported for posterior lumbar interbody fusion (PLIF) [14,15]. BGS-7 spacers exhibited fusion rates, clinical outcomes, and adverse events comparable to those of titanium cage [14,15]. So far, only one study reported the feasibility of BGS-7 in ACDF surgery [16]. However, more studies should be conducted to address the issue. 

**Figure 1:**
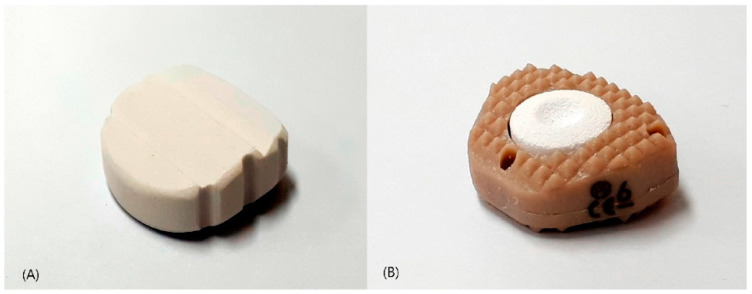
Photographs of implants: (**A**) CaO-SiO_2_-P_2_O_5_-B_2_O_3_ bioactive glass ceramic spacer (**B**) polyetheretherketone cage filled with hydroxyapatite/β-tricalcium phosphate.

The objective of this study is to present a report on the safety and effectiveness of BGS-7 when utilized as an interbody spacer for ACDF, as depicted in Figure 1A. In this study, the BGS-7 spacer was implanted in patients with cervical degenerative disease who required one- or two-level ACDF, to compare its safety and effectiveness to those of PEEK cages filled with a mixture of hydroxyapatite (HA) and β-tricalcium phosphate (β-TCP) (Figure 1B). Thus, the aim of this study was twofold: firstly to assess the bone fusion rates and clinical outcomes of the BGS-7 interbody spacer compared to the PEEK cage filled with HA and β-TCP; and secondly, to ascertain the feasibility of substituting the cage with the BGS-7 interbody spacer.

## 2. Materials and Methods

### 2.1. Study Design and Participants

This is a prospective, randomized, non-inferiority trial in patients with cervical degenerative disease who underwent ACDF using a BGS-7 interbody spacer or a PEEK cage. The study protocol was approved by the institutional review board of our hospital (E-1505/298-001) and registered at ClinicalTrials.gov as NCT02425514 (accessed on 4 January 2019). Written informed consent was obtained from all the enrolled participants. All enrolled participants were randomly assigned to either the BGS-7 group (Group N) or the PEEK cage group (Group C). 

The inclusion criteria were participants aged between 20 and 80 years who were expected to undergo one- or two-level ACDF for degenerative cervical spine diseases between June 2015 and August 2016. We excluded participants who were diagnosed with cervical spine fracture, infection, or malignancies at the surgical level, severe osteoporosis (below −3.5 T-score on bone densitometry), and other disorders that were considered inappropriate for participation. 

### 2.2. Randomization and Follow-Ups

After evaluating the baseline characteristics, the participants were allocated to Group C or Group N following randomization lists generated by the program nQuery Advisor (Statistical Solutions, Boston, MA, USA). The randomization lists were accessible to a researcher who was not involved in the trial to maintain the concealed allocation. In this single-blinded trial, the participants were blinded to the group to which they were assigned. All randomized participants underwent surgery by a single orthopedic spine surgeon (J.S.Y) at our tertiary institution.

The study subjects were subjected to a minimum of 12 months of active follow-up. The primary and secondary outcomes were gathered by an independent researcher during in-person hospital visits or telephone communication. The assessment of outcomes was conducted at baseline and at follow-up intervals of 3, 6, and 12 months. 

In the initial protocol, the primary outcome measure was the fusion rate on computed tomography (CT) 6 months postoperatively. However, the fusion rate on CT at 6 months postoperatively was lower than expected, and the follow-up period was extended by 6 months to analyze the fusion rate on CT at 12 months.

### 2.3. Interventions: Anterior Cervical Discectomy and Fusion

The standard Smith–Robinson approach to the cervical spine was performed through a transverse incision. Following the removal of the disc material, endplates were meticulously prepared utilizing a quadrangular curette and other pertinent surgical instruments. The neural structures were subjected to decompression, with or without uncoforaminotomy, as deemed necessary. The intradiscal cartilaginous tissues were meticulously removed; however, the endplates were left undecorticated. A PEEK cage or BGS-7 spacer was gently implanted according to predetermined randomized group allocations. The selection of spacer height was contingent upon the intraoperative assessment conducted through the utilization of spacer trials. Rigid anterior platescrew fixation using Atlantis Vision^®^ Elite (Medtronic, Memphis, TN, USA) or TriSecure^TM^ (CG Bio, Inc., Seongnam, Republic of Korea) was performed in all participants.

### 2.4. Outcomes and Measurements

Baseline characteristics collected by a clinical research assistant otherwise blinded to the study included demographic data, medical comorbidities using the Charlson Comorbidity Index (CCI), and American Society of Anesthesiologists (ASA) physical status classification. Patient-reported outcomes (PROs) were also collected from the participants preoperatively and at the 3-, 6-, and 12-month postoperative follow-ups.

The primary outcome measure was the fusion rate on CT at the 12-month postoperative follow-up. Secondary outcome measures were the fusion rate on dynamic radiographs and changes in PROs. The PROs included the pain visual analog scale (VAS) score for neck and upper extremity pain, and scores from the neck disability index (NDI), European Quality of Life-5 Dimensions (EQ-5D), and 12-item short-form health survey revised form (SF-12v2) for health-related quality of life (HRQOL). Surgery-related complications have been reported previously. 

For the primary outcome, fusion was defined as the presence of bone bridging and/or a lack of radiolucency at the graft-vertebral junction on coronal or sagittal CT images using a 3D surgical simulation software (Vworks v.4.0; Cybermed, Inc., Reston, VA, USA). Coronal and sagittal images were reconstructed using 1.0-mm-interval axial CT scan images of the cervical spine. Fusion status was assessed based on the agreement of three orthopedic surgeons with 9, 7, and 6 years of experience, who were not involved in providing direct patient treatment. For secondary outcomes, fusion was defined as an average interspinous distance of <1 mm on dynamic radiographs at the operated level at 12-month postoperative follow-up using PACS (Infinitt, Bracknell, Berkshire, UK) [17]. All measurements were performed by three orthopedic spine surgeons who were not involved in providing direct patient treatment.

The patients’ level of pain was evaluated through the utilization of a 10-cm VAS to measure both neck and arm pain. The NDI is a specialized questionnaire designed to assess the degree of disability associated with neck pain. The questionnaire utilized a scoring system where higher scores are indicative of more severe levels of disability. The EQ-5D is a widely employed tool for assessing HRQOL, wherein elevated scores correspond to superior HRQOL. The SF-12v2 is a health survey used to measure HRQOL. This survey was partitioned into two summary scores: the physical component summary score (SF12-PCS) and the mental component summary score (SF12-MCS). The scores were designed to reflect the respondent’s HRQOL, with higher scores indicating a superior HRQOL. The term, “complications” were defined as intraoperative or postoperative adverse events, including but not limited to incidental durotomy, wound infection, reoperation, and readmission specifically for surgery-related reasons.

### 2.5. Statistical Analysis

For the primary outcome analysis, we calculated that a sample size of 76 participants would provide at least 80% power to show the non-inferiority of BGS-7 spacer relative to PEEK cage with a one-sided alpha level of 0.05, and a non-inferiority margin of 15% for the 6-month fusion rate of ACDF with cervical PEEK cage, assuming a 30% dropout rate at 6 months [7]. 

Our primary analysis, the per-protocol (PP) strategy, included patients who underwent surgery and completed a 12-month follow-up period. Continuous variables are presented as mean and standard deviation (SD), whereas categorical variables are presented as numbers and percentages (%). For the primary outcome, 12-month fusion rates with one-sided 95% confidence intervals (CIS) for the intergroup differences were calculated. Non-inferiority of BGS-7 spacer was confirmed if the lower limit of the 95% CI of the fusion rate at 12-month was higher than the predefined non-inferiority margin of −15% (fusion rate of group C—group N ≥ 15%). To analyze serial measurements of clinical outcomes (VAS pain score of the neck and upper extremities, NDI score, SF12-PCS score, SF12-MCS score, and EQ-5D value), we used a linear repeated-measures mixed model. Time was defined as a categorical variable, including 3, 6, and 12 months. We also analyzed intervention–time interactions to examine interbody spacer effects and intergroup differences during the 12-month follow-up period using a linear repeated-measures mixed model. Other secondary outcomes were analyzed using Student’s *t*-test (continuous variables) or Fisher’s exact test (categorical variables). 

Interobserver and intraobserver agreements were assessed using Cohen’s kappa value (95% CI) according to Landis et al.’s method (0.01–0.20: slight agreement, 0.21–0.40: fair agreement, 0.41–0.60: moderate agreement, 0.61–0.80: substantial agreement, and 0.81–1.00: nearly perfect agreement) [18]. Three reviewers analyzed the CT scans and dynamic radiographs after a 4-week interval to assess the intra-rater agreement. Differences in the radiographic results in the assigned fusion evaluation for consensus reading were resolved through a joint review of the CT images with a unanimous decision.

All tests were conducted using Stata/MP 15.1 (StataCorp LLC, College Station, TX, USA). Statistical significance was defined as a two-sided *p*-value < 0.05, except for the *p*-value from the one-sided non-inferiority test.

## 3. Results

### 3.1. Participant Characteristics

**Figure 2:**
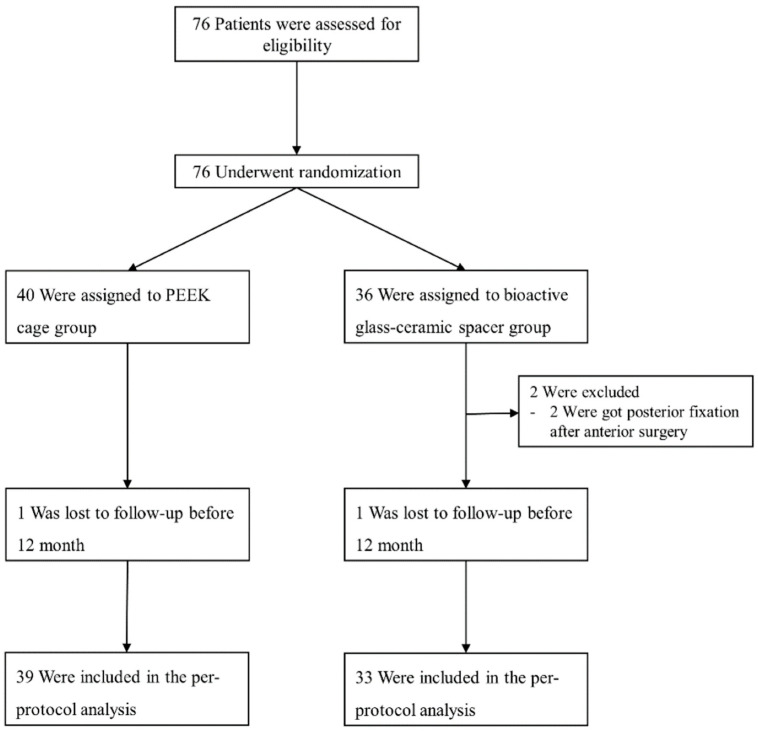
Enrollment, randomization, treatment, and follow-up flow diagram.

A total of 76 participants were assessed for eligibility, and all of them provided consent to undergo randomization into one of the treatment groups. The allocation of participants was conducted randomly, resulting in Group C (n = 40) or Group N (n = 36). None of the randomized treatment strategies exhibited any crossover. Two subjects were excluded from group N because they underwent posterior fixation after anterior surgery. Two subjects were excluded from the PP analysis due to loss of follow-up postoperatively (one patient in group C and one patient in Group N). Consequently, the final analysis comprised a total of 74 patients. The flow diagram of the study and follow-up is depicted in Figure 2.

Table 1 displays the baseline characteristics of the participants in each group. There were no significant differences between the two groups at baseline in terms of clinical and radiographic characteristics (all *p* > 0.05).

### 3.2. Primary Outcome at 12-Month Follow-Up

**Figure 3:**
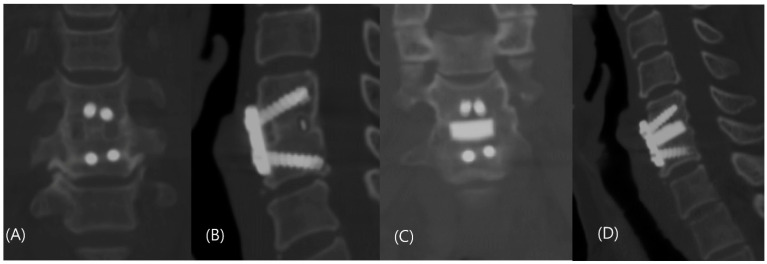
Coronal and sagittal computed tomography scan taken 12 months postoperatively showed complete fusion of (**A**,**B**) group C and (**C**,**D**) group N.

On follow-up CT scans at 12 months postoperatively, fusion was achieved in 29 participants in group C (74.4%; 29/39) and 27 participants in group N (81.8%; 27/33). The risk difference between the two groups was 7.4% (95% CI: −11.5% to +26.5%). The noninferiority of the BGS-7 spacer was confirmed (Table 2 and Figure 3). The Kappa value for interobserver reliability was 0.774, indicating substantial agreement. The Kappa values for intraobserver reliability were 0.891, 0.818, and 0.813 for each rater, respectively, indicating nearly perfect agreement.

### 3.3. Secondary Outcomes and Adverse Events

On follow-up dynamic radiographs at 12-month postoperative follow-up, fusion was achieved in 28 participants in group C (73.7%; 28/38) and 27 participants in group N (78.1%; 25/32), showing no significant difference between the groups (*p* = 0.666). The Kappa value for interobserver reliability was 0.618, indicating substantial agreement. The Kappa values for intraobserver reliability were 0.718, 0.856, and 0.671 for each rater, indicating substantial to nearly perfect agreement.

**Figure 4:**
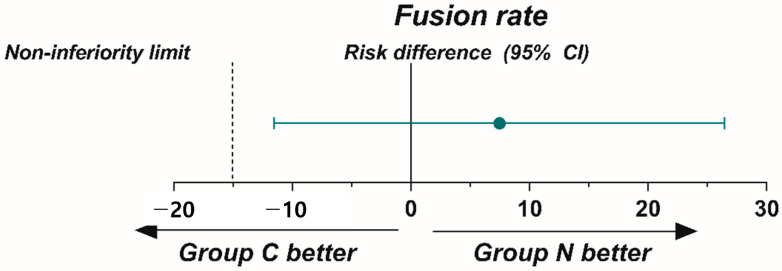
The graph illustrates the absence of significant disparity in in fusion rate between the two groups 12-months postoperatively. The dashed line denotes a threshold for non-inferiority, set at a negative deviation of 15% from the reference value. Confirmation of the non-inferiority of bioactive glass ceramic spacer is achieved when the upper limit of the one-sided 95% confidence interval falls below the predetermined margin.

The linear mixed model showed no significant intervention effect on neck and upper extremity pain, disability, or HRQOL during the 12-month follow-up period (Figure 4). There were also no intergroup differences in the VAS neck pain, VAS upper extremity pain, NDI, EQ-5D, SF12-PCS, and SF12-MCS scores at the 3-, 6-, and 12-month follow-ups (Table 3). There were no surgery-related complications, such as incidental durotomy, hematoma, prevertebral swelling, infection, thromboembolic events, pneumonia, stroke, cardiac arrest, or neurological damage.

## 4. Discussion

The attainment of intervertebral fusion is a crucial aspect in achieving a favorable long-term outcome in ACDF. Various techniques have been employed for intervertebral fusion in the context of ACDF, including autogenous bone grafts from the iliac crest, allografts, cages filled with bone graft materials, and ceramic spacers. The use of intervertebral cages has gained popularity in ACDF surgery because they are designed to provide structural support between the vertebral bodies, maintain the intervertebral disc space, and facilitate intervertebral fusion within and around the cage. This has been substantiated by various studies [1,2,3,4,5,6]. This study, which included 76 participants with cervical degenerative disorders, was designed to evaluate and compare the radiographic and clinical outcomes between the BGS-7 spacer and PEEK cage. The study was conducted as a single-center trial, and the follow-up period was 12 months postoperatively. The aim was to confirm the non-inferiority of the BGS-7 spacer in comparison to the PEEK cage. Moreover, the clinical outcomes and adverse events associated with the BGS7 spacer were comparable to those associated with the PEEK cage.

PEEK is a commonly utilized synthetic material among various options for ACDF. PEEK exhibits an elastic modulus similar to that of human bone, resulting in reduced cage subsidence and improved load distribution between the cage and bone [19,20,21,22]. The quality of radiolucency is a desirable characteristic when assessing the success of postoperative fusion status through radiographs as well as reducing the impact of implant artifacts on postoperative CT or MRI scans. PEEK, despite its utility, lacks the necessary biological properties for promoting bone regeneration, namely osteoconduction and osteoinduction [22]. In order to address this constraint, PEEK cages have been augmented with various substances including autologous local bone, HA, β-TCP, and DBM [1,5,21,22]. 

The recently introduced BGS-7 spacer has been reported to have a relatively high mechanical strength compared to the PEEK cage [10,16]. The composition of BGS-7 was CaO 41.79, SiO2 35.82, P2O5 13.93, B2O3 0.5, CaF2 1.99, and MgO 5.97 (weight%). The compressive strength analysis of BGS-7 spacer, titanium, and PEEK cages of identical size revealed that the BGS-7 spacer exhibited strength that was 4 and 1.3 times greater than those of the the PEEK and titanium cages, respectively [10]. The material in question is recognized for its elevated biocompatibility and favorable osteoconductivity, and is deemed to be biologically safe [9,10,11,12,13,14]. According to Lee et. al, it has been reported that BGS-7 facilitates the process of osteoblastic differentiation in human mesenchymal stem cell [12]. The in vivo model showed superior bone bonding to neighboring bones in comparison to HA [12]. Additionally, coating the surface with the dense cylindrical shaped specimen of BGS-7 stimulated osteointegration of implants [12]. Studies on repetitive toxicity has indicated that there are no instances of adverse reaction related to BGS-7 following high-dose intravenous injections of aqueous extracts of BGS-7 for a period of 90 days [13]. Furthermore, due to its limited bioabsorption, the potential for unfavorable tissue or systemic responses is exceedingly minimal [14]. However, the chronic and enduring toxicity and unfavorable consequences of the BGS-7 remained undisclosed. Nevertheless, it could be considered a possible alternative graft for ACDF.

The findings of our study indicated that the fusion rate observed in Group N was 81.8 % as determined by the 12-month postoperative CT scan. The fusion rate of Group N at 12-month based on dynamic radiographs was found to be 78.1%. The fusion rates of Group N, as evaluated by CT scan and dynamic radiographs, exhibited a tendency towards higher values as compared to those of group C. Specifically, the fusion rates for Group C were 74.4% and 73.7%, respectively, but a difference has not been proven statistically. In a previous clinical trial involving the use of BGS-7 spacer in PLIF, it was observed that the fusion rate of the group treated with BGS-7 spacer was 89.7% in a 12-month CT scan and 90.6% in a 48-month CT scan. These fusion rates were found to be comparable to those of titanium cages [14,15]. The observed variance in fusion rate in comparison to prior studies on posterior lumbar interbody fusion (PLIF) is thought to stem from dissimilarities in the graft volume of autologous local bone or variations in the contact surface area between the spacers and endplates. In contrast to the contact surface area of the bone graft materials contained within the polyetheretherketone (PEEK) cage, the BGS-7 spacer exhibited a greater contact surface area, thereby yielding a more extensive fusion region [16]. Furthermore, BGS-7 spacer’s direct chemical bonding ability with the bone improves the osseointegration of the spacer into the adjacent endplates. The broad fusion area efficiently disperses the pressure on the spacers in interbody fusion, leading to a reduced incidence of subsidence [23,24,25,26,27]. The study findings indicate that while there was no statistically significant difference in fusion rates between the PEEK cage with HA and β-TCP and the BGS-7 spacer, a wider contact surface area may lead to higher fusion rates in the BGS-7 group. Furthermore, the absence of mechanical complications, such as spacer breakage or endplate subsidence, can be attributed to the superior mechanical strength of BGS-7 in comparison to the PEEK cage. This mechanical strength of BGS-7 appears to have a negligible impact on subsidence, based on the interpretation of the current data. 

**Figure 5:**
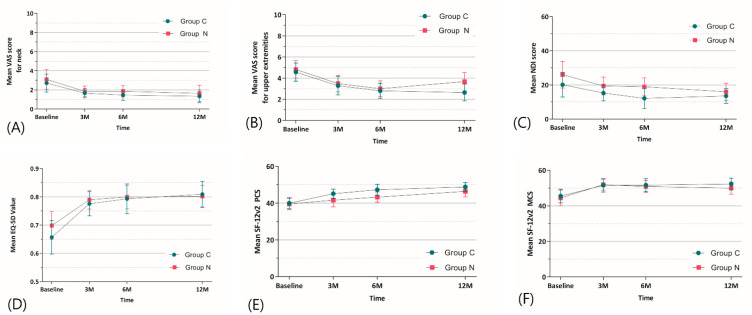
Changes in secondary outcomes between the two groups during the 12-month follow-up period. (**A**) Changes in mean VAS neck pain score, ranging from 0 (no pain) to 10 (worst pain). (**B**) Changes in mean VAS upper extremity pain score, ranging from 0 (no pain) to 10 (worst pain). (**C**) Changes in mean NDI score, ranging from 0 (no disability) to 100 (high disability). (**D**) Changes in mean EQ-5D value, ranging from 0 (worst quality of life) to 100 (best quality of life). (**E**) Changes in mean SF-12v2 PCS and (**F**) MCS score, with higher scores indicating better HRQOL. Error bars indicate 95% confidence intervals. VAS, visual analog scale; NDI, Neck disability index; EQ-5D, European Quality of Life-5 Dimensions; SF-12, 12-item short form health survey; PCS, physical component summary; MCS, mental component summary.

In the clinical results, both groups showed significant improvement in terms of neck and arm pain, disability in patients’ daily lives, and HRQOL compared with the preoperative status, with no significant intergroup differences (Figure 5). In the safety assessment results of this trial, there were no adverse events due to the medical device during 12-month postoperative follow-up. However, considering that this is a new material, a longer follow-up period is needed. 

To the best of our knowledge, this is the first trial to investigate the radiographic and clinical outcomes of the BGS-7 spacer and the PEEK cage at 12 months postoperatively. However, this study has several limitations. First, this trial had a small sample size, which prevented generalized conclusions on the potential differences between the two interventions. The preliminary calculation of the sample size indicated that 38 individuals would be necessary in every group, accounting for a 30% attrition rate. During the final follow-up assessment, an adherence rate exceeding 97% was noted. Thus, it was deduced that the trial exhibited a superior statistical power in comparison to the anticipated outcome. Secondly, the 12 month end-point of our trial was insufficient to evaluate the long-term merits and demerits of this new device utilized in ACDF surgery. Furthermore, it should be noted that the clinicians conducting the trial were not subject to blinding with regards to the allocation of intervention. However, the study employed an independent researcher to conduct the clinical outcome measures, who was blinded to the allocation. Additionally, the participants were also blinded to their allocation. Consequently, it is improbable that the utilization of a subject–assessor, double-blind design had any impact on the outcomes. The utilization of multiple comparisons in the examination of secondary outcomes resulted in an elevated likelihood of type 1 errors. Given this possible constraint, it is advisable to conduct an analysis of the statistical impact of interventions on secondary outcomes.

## 5. Conclusions

In this prospective, randomized controlled trial of participants who underwent ACDF surgery, the results confirmed the non-inferiority of the BGS-7 spacer to the PEEK cage at 12 months postoperatively. Our findings confirmed the non-inferiority of the BGS-7 spacer compared to the PEEK cage and revealed no differences in clinical outcomes between the two groups during the follow-up period. Therefore, in patients undergoing ACDF, the BGS-7 ceramic spacer is considered a good alternative graft option to PEEK cages. 

## Figures and Tables

**Table 1:** Characteristics of the participants at baseline.

Characteristic	Control Group (n = 40)	Study Group (n = 36)
Age (years)	55.0 ± 11.3	54.4 ± 10.6
Male/Female *	27/13	25/11
BMI (kg/m^2^)	24.7 ± 2.3	24.4 ± 2.3
CCI score	1.7 ± 1.6	1.6 ± 1.5
ASA score	1.5 ± 0.6	1.4 ± 0.5
Smoking status, n (%) *		
Non/Ex-smoker	21 (52%)	19 (53%)
Current smoker	19 (48%)	17 (47%)
Alcohol consumption, n (%) *		
None	10 (25%)	12 (33%)
≥1 drink/month	30 (75%)	24 (67%)
VAS for neck pain	2.8 ± 2.9	3.0 ± 2.9
VAS for arm pain	4.6 ± 2.7	4.9 ± 2.3
NDI	21.0 ± 22.8	25.1 ± 21.9
EQ-5D	0.649 ± 0.189	0.695 ± 0.139
SF-12		
PCS	39.7 ± 9.3	38.9 ± 8.7
MCS	45.0 ± 12.1	43.9 ± 12.3
Diagnosis, n (%) *		
Myelopathy	22 (55%)	17 (47%)
Radiculopathy	17 (43%)	17 (47%)
Myeloradiculopathy	1 (2%)	2 (6%)
Approach side, n (%) *		
Right	22 (55%)	19 (53%)
Left	18 (45%)	17 (47%)
Operation level, n (%) *		
C3-4	5 (10%)	4 (9%)
C4-5	10 (20%)	9 (19%)
C5-6	20 (40%)	19 (40%)
C6-7	12 (24%)	12 (26%)
C7-T1	4 (6%)	3 (6%)

Data are presented as mean ± standard deviation. * Data are presented as number of patients. BMI, body mass index; CCI, Charlson Comorbidity Index; ASA, American Society of Anesthesiologist; VAS, visual analog scale; NDI, neck disability index; EQ-5D, European Quality of Life-5 Dimensions; SF-12, 12-item short form health survey; PCS, physical component summary; MCS, mental component summary.

**Table 2:** Fusion rate of each group at 12 months postoperatively.

	Group C	Group N	*p*-Value *	Risk Difference (95% CI)
Fusion rate on CT ^†^	74.4%	81.8%		7.4% (−11.5% to 26.5%)
Fusion rate on dynamic radiographs (ISM < 1 mm) ^‡^	73.7%	78.1%	0.666	

CI, confidence interval; CT, computed tomography; ISM, interspinous motion. * Chi-square test was used. ^†^ Fusion rate on CT is the primary outcome of this study, and the risk difference was calculated to show non-inferiority. **^‡^** The fusion criteria of interspinous motion (ISM) on dynamic radiographs was defined as ISM < 1 mm at the arthrodesis level.

**Table 3:** Secondary outcomes for both groups at the 12-month postoperative follow-up.

Variables	Group C	Group N	Mean Difference (95% CI)	*p*-Value
VAS neck				
3 months	1.67 ± 1.51	1.85 ± 1.52	−0.18 (−0.90–0.53)	0.722
6 months	1.46 ± 1.70	1.85 ± 1.68	−0.39 (−1.18–0.41)	0.449
12 months	1.33 ± 2.06	1.64 ± 2.40	−0.30 (−1.35–0.74)	0.554
Overall intervention effect *	NA	NA	NA	0.988
VAS upper extremities				
3 months	3.28 ± 2.69	3.48 ± 2.21	−0.20 (−1.37–0.97)	0.723
6 months	2.79 ± 2.21	3.00 ± 2.11	−0.21 (−1.23–0.82)	0.720
12 months	2.64 ± 2.48	3.67 ± 2.45	−1.03 (−2.19–0.14)	0.073
Overall intervention effect *	NA	NA	NA	0.535
NDI				
3 months	15.26 ± 14.36	19.39 ± 14.83	−4.13 (−11.01–2.74)	0.307
6 months	12.14 ± 17.92	18.90 ± 14.74	−6.76 (−14.56–1.05)	0.095
12 months	13.57 ± 13.53	15.97 ± 14.11	−2.40 (−8.91–4.11)	0.553
Overall intervention effect *	NA	NA	NA	0.782
EQ-5D				
3 months	0.775 ± 0.132	0.790 ± 0.093	−0.014 (−0.069–0.040)	0.665
6 months	0.793 ± 0.163	0.799 ± 0.117	−0.007 (−0.074–0.061)	0.842
12 months	0.809 ± 0.140	0.801 ± 0.111	0.008 (−0.053–0.068)	0.816
Overall intervention effect *	NA	NA	NA	0.591
SF12-PCS				
3 months	45.16 ± 7.92	41.64 ± 10.41	3.52 (−0.79–7.83)	0.089
6 months	47.28 ± 9.29	43.28 ± 7.98	4.01 (−0.11–8.12)	0.053
12 months	48.80 ± 7.54	46.41 ± 8.47	2.38 (−1.38–6.14)	0.259
Overall intervention effect *	NA	NA	NA	0.444
SF12-MCS				
3 months	51.52 ± 11.71	51.89 ± 8.84	−0.36 (−5.31–4.59)	0.887
6 months	51.62 ± 44.45	50.88 ± 9.45	0.74 (−4.23–5.71)	0.773
12 months	52.32 ± 10.24	49.96 ± 9.74	2.35 (−2.37–7.08)	0.357
Overall intervention effect *	NA	NA	NA	0.879

95% CI, 95% confidence interval; VAS, visual analog scale; NA, not available; NDI, neck disability index; EQ-5D, European Quality of Life-5 Dimensions; SF-12, 12-item short form health survey; PCS, physical component summary; MCS, mental component summary. Data are presented as mean ± standard deviation. * *p*-value is from linear mixed models for repeated measures compared between interventions during 12-month follow-up period.

## Data Availability

Data related with this study can be provided by the corresponding author upon request.
